# Mommy has to go

**DOI:** 10.1007/s11606-025-09658-5

**Published:** 2025-07-09

**Authors:** Sarah S. Fittro

**Affiliations:** Vanderbilt University Medical Center, Nashville, TN USA

Mom was awake all night

Admitting to the wards

Soothing her newborn

Mom was late

For morning report

To get home for bedtime

Mom worried

About her patient’s increasing need for oxygen

About her baby’s need for his mother

Mom ached

In her back from long rounds

In her heart for her child

Mom missed

The conference deadline

Baby’s first steps

Mom couldn’t sleep

Were the orders correct?

Why does my baby have a fever?

Mom wondered

Did stress cause the miscarriage?

Was this worth all the training?

Mom cried

As her patient took their last breath

As her baby’s first year flew by

Mom nursed

Her patients back to health

Her baby into life

Mom loved

Her work in medicine

Her baby at home

Mommy has to go

She has patients to save and a baby to hold

